# Influence of Polypropylene Fibre Factor on Flowability and Mechanical Properties of Self-Compacting Geopolymer

**DOI:** 10.3390/ma14175025

**Published:** 2021-09-02

**Authors:** Bei-chen Pu, Bin Liu, Li Li, Wei Pang, Zhangrun Wan

**Affiliations:** 1 School of Highway, Chang’an University, Xi’an 710064, China; 2012021053@chd.edu.cn; 2 CSCEC AECOM Consultants Co. Ltd., Lanzhou 730000, China; pw_bridge@126.com; 3 College of Water Resources and Architectural Engineering, Northwest A&F University, Yangling 712100, China; drlili@nwafu.edu.cn (L.L.); liyu2188@nwafu.edu.cn (Z.W.); 4 State Key Laboratory of Green Building Materials, China Building Materials Academy, Beijing 100024, China

**Keywords:** polypropylene fibre, fibre factor, flowability, mechanical properties, geopolymer, self-compacting

## Abstract

The possibility of using geopolymer instead of Portland cement could effectively reduce carbon dioxide emissions from cement manufacturing. Fibre-reinforced self-compacting geopolymers have great potential in civil engineering applications, such as chord member grouting for concrete-filled steel tubular truss beams. However, to the best of the authors’ knowledge, the quantitative relationship between FF and the properties of the fibre-reinforced geopolymer has been rarely reported. In this research, 26 groups of mixtures were used to study the influence of the polypropylene fibre factor (FF) on the flowability and mechanical properties and also the compactness of the fibre-reinforced self-compacting geopolymer. At the same volume fraction, geopolymers with long fibres present worse flowability than those having short fibres due to the easier contacting of long fibres. By growing the FF the influence of fibre addition on the V-funnel flow rate is more significant than the slump spread. This can be ascribed to the consequence of fibre addition and friction by the V-funnel which estimates the restrained deformability. For FF lesser than critical factor Fc = 100, influence of fibres is negligible and fibres are far apart from each other and, thus, they cannot restrict cracking under load and transfer the load to improve the mechanical properties. For FF between the Fc = 100 and density factor Fd = 350, a noteworthy enhancement of mechanical properties was obtained and the geopolymer was still adequately workable to flow by weight of self, without any symbols of instability and fibre clumping. Under this condition, better fibre dispersal and reinforcing productivity can lead to better hardened properties. For FF higher than Fd = 350, fibres tend to come to be entwined together and form clumping resulting from the fibre balling, resulting in worse hardened properties. This research offers a sensible basis for the application of the workability regulator of the fresh properties of fibre-reinforced geopolymer as an operative way to basically obtain ideal mechanical properties.

## 1. Introduction

It is known to all that Portland cement is the most widely used binding material in concrete in the world. One t-carbon dioxide is emitted for every ton of Portland cement manufactured, so it is significant to find green cementing materials [1]. The possibility of using geopolymer instead of Portland cement could effectively reduce carbon dioxide emissions from cement manufacturing [2,3]. Moreover, the raw materials for the preparation of geopolymer can be industrial wastes, for instance fly ash, slag, silica fume, waste concrete and so on. The consumption of industrial wastes in geopolymer is remarkable from economic and environment standpoints [4]. Geopolymers illustrate greater mechanical properties and resistance to high temperature, sulphates, and acids than Portland cement-based composites [5]. Nevertheless, as Portland cement-based composites, geopolymers display brittle failure resulting from their low tensile strength that could enforce some restrictions in potential engineering application. Conventionally, Portland cement-based composites are reinforced by the inclusion of fibres manufacturing concrete with a toughness performance [6,7,8,9,10,11,12,13]. Hence, different kinds of fibres have been used to reinforce geopolymers [14,15,16,17,18].

This reinforcement of fibres on cementitious composites is greatly dependent on the length-to-diameter ratio (l/d) and the content of fibres [19]. Most research in the literature reveals that the mechanical properties of fibre-reinforced cementitious composites rise with the fibre factor (FF), specifically the product of the aspect ratio and the volume fraction of the fibres [20,21,22,23]. In spite of the benefits of fibres strengthening, fibres reduce the flowability of fresh composites, which can be an impediment for a good uniformity, solidity and mechanical property of the hardened composites. This damaging influence is worsened with the enlarged length-to-diameter ratio and amount of fibres. For example, the slump of fibre-reinforced concrete declines with the growth of FF [24,25]. According to the theoretical model proposed by Krieger and Dougherty [26], the relationship between FF and the flowability of steel, polypropylene (PP) and glass fibre-reinforced cementitious composite was studied [27,28,29,30]. Cao et al. [31] and Si et al. [32,33] studied the influence of FF of the polyvinyl alcohol (PVA) fibre and steel-PVA hybrid fibre on the rheological and mechanical properties of mortar. In these researches, it is illustrated that there exists a critical FF of fibres above which the cementitious composites could not flow anymore relying solely on self-weight, and fibres are apt to the occurrence of clumps or balls.

Obviously, the fibres also affect the flowability and mechanical properties of the geopolymer [34,35]. Fibre self-compacting geopolymers have great potential in civil engineering applications, such as chord grouting for concrete-filled steel tubular composite truss beams. However, to the best of the authors’ knowledge, the quantitative relationship between FF and the properties of fibre-reinforced geopolymer has been rarely reported. In addition, the critical FF of fibres in geopolymer has never been revealed. Hence, the objective of this research is to study the influence of PP FF on the flowability, mechanical properties and also the compactness of the fibre-reinforced geopolymer. Additionally, the critical and dense values of FF concerning both the flowability and mechanical properties of geopolymer are cleared. The investigation on PP fibre-reinforced self-compacting geopolymer is still very inadequate. The discoveries in this paper offer a sensible groundwork for the application of a workability control of fresh properties of geopolymer as an operative way to attain optimal mechanical properties.

## 2. Fibre Factor Theoretical Methods

The influence of fibre on the rheological and mechanical properties of hardened composite is governed by lots of factors, such as fibre volume fraction (*φ*), rigidity, length (*l*) and diameter (*d*). Related research presents that FF is the most useful factor to describe the workability and mechanical properties of fibre-reinforced cementitious composites.

Single fibre factor is obtained by multiplying fibre volume fraction and fibre aspect ratio. The practical significance of fibre factor is derived from Krieger and Dougherty’s equation [26] and Philipse’s random contact equation [36]. Fibre-reinforced cement-based composites are simplified as a mixture of suspended rigid spherical particles and suspended liquid with thin rods. In this system, Krieger and Dougherty proposed a method to quantify the effect of fibre particle content on the viscosity of the system by using Equation (1).

(1) $$\eta_{r}={\left(1-\frac{\varphi }{\varphi_{max}}\right)}^{-\varphi_{max\left[\eta \right]}}\;$$

where $\eta_{r}$ is the relative suspension degree, that is, the suspension viscosity divided by the viscosity of the suspension fluid;

$\left(\eta \right)$ is the characteristic viscosity of a basic characteristic parameter. The typical value of rigid spherical monodisperse suspension is 2.5;

φ is the volume fraction of solid particles, and $\varphi_{max}$ is the maximum particle filling fraction, which is about 0.605 under high shear stress.

It can be seen from Equation (1) that the relative suspension viscosity $\eta_{r}$ and $\varphi /\varphi_{\max}$ are closely related. According to the literature [27,28,29,30], Krieger Dougherty equation is suitable for the fresh paste of fibre-reinforced concrete, and the properties of fresh paste of fibre-reinforced concrete can be divided into three stages according to the equation $\varphi /\varphi_{\max}$ value. In the first stage, due to the value of $\varphi /\varphi_{\max}$ being low, there are only a few fibres in the suspension, and these fibres are far away from each other, so the probability of overlapping and clustering is very low, which almost does not affect the workability and mechanical properties of fibre-reinforced concrete. With the increase of $\varphi /\varphi_{\max}$, the volume fraction of fibre in the suspension increases until it exceeds the certain value of $\varphi /\varphi_{\max}$. Additionally, the fibres begin to contact each other. At this stage, the fresh slurry is considered to be a semi diluted system, and the probability of contact between fibres increases with the increase the value of $\varphi /\varphi_{\max}$. In previous experiments [27,28,29,30], when there are enough fibres, the free water in the matrix for lubrication is less and less, and the fluidity of fresh paste of fibre-reinforced concrete is reduced. However, with the increase of the number of fibres, the bridging effect is more obvious, and the mechanical properties of hardened products are improved significantly. At the end of this stage, the performance of fibre-reinforced concrete reaches the best state at the same time. At this time, the volume fraction of the added fibre is in the range of $\varphi_{d}$. $\varphi_{d}$ is the critical or maximum fibre content required in fibre-reinforced concrete. When the volume fraction of fibre exceeds $\varphi_{d}$, the results showed that the probability of fibre clustering in the process of stirring greatly increased. According to previous studies [27,28,29,30], the deterioration degree of processing performance and mechanical properties of fibre-reinforced concrete in this area has several ranges. The value of $\varphi /\varphi_{\max}$ is very important for the study of the performance of fibre-reinforced concrete. However, due to the softness of chemical fibre, it is difficult to measure the maximum filling rate of solid particles containing chemical fibre. Therefore, in order to quantify the fibre filling state depending on the contact number (*c*), where *r* is the fibre aspect ratio, the random contact Equation (2) is a model to simulate the possibility of contact between any thin rod and adjacent thin rod at any volume fraction.

(2) $$r\left[\frac{4}{r}+\frac{3r}{3r+2}\right]=c$$

For large aspect ratio, the equation is simplified as follows:

(3) φr≅cr≫1

Equation (3) shows that the fibre factor *F* can finally approach a constant at large aspect ratio. Similarly, for the maximum filling rate of fibre, the equation can be converted to Equation (4)

(4) $$\varphi_{max}r≅c_{max}r\gg 1$$

Equation (5) gives the simple mathematical calculation of Equations (1) and (4)

(5) $$\eta_{r}={\left(1-\frac{\varphi r}{c_{max}}\right)}^{\frac{-c_{max}\left[\eta \right]}{r}}$$

From Equation (5), it can be seen that *F* = φr is the most important factor affecting the relative viscosity of the suspension, not φ/φ_max_.

In addition, when $c_{max}$ is a constant, due to the efficiency reaching $\varphi /\varphi_{\max}$, according to the value range of *F*, the workability of fresh fibre-reinforced paste can be divided into three states; these are the dilution state, semi consolidation dilution state and consolidation state. It can be seen from the above theory that *F* is indeed the key factor to be considered when adjusting the performance of fibre-reinforced composites and carrying out mix design. According to specific fibre factors $F_{c}$ and $F_{d}$ in this study, the practical significance of PP fibre-reinforced geopolymer was studied.

## 3. Materials and Testing

### 3.1. Properties of Raw Materials

Silica fume (SF), fly ash (FA) and slag (SL) were bought from the Henan Dingnuo Purification Material Co., Ltd., Zhengzhou, China, Shaanxi Weihe Power Plant in Xianyang, China, and Xi’an Delong Powder Engineering Materials Co., Ltd., Xi’an, China, respectively. The chemical compositions of mineral materials were tested by XRF, as presented in Table 1. The alkaline activator with modulus (M = n(SiO_2_) / n(Na_2_O)) of 1.5 was compounded by sodium silicate and sodium hydroxide. Sodium silicate has molar ratio SiO_2_/Na_2_O of 3.22, produced by Changlong Water Glass Factory, Gongyi, China. NaOH (analytically pure, Guangdong Guanghua Sci-Tech Co., Ltd., Shantou, China) was employed to adjust the modulus of alkaline activator. Then, the fresh paste was used to test rheological behaviour. Marketable PP fibres with 6 and 12 mm lengths were also used. Dimensions and mechanical properties of the PP fibres are shown in Table 2. Water/binder (w/b) ratio was defined as the mass ratio of water (total mass of water in sodium silicate solution and water added) to mineral materials (FA, SL and SF). The w/b ratio of all the fresh geopolymer pastes was set at 0.3 and the alkali content was fixed as 7%.

### 3.2. Mix Proportion

In this research, 26 groups were planned in which two lengths of PP fibres at various volume fractions (0, 0.3, 0.6, 0.9, 1.2, 1.5 and 1.8% by volume of geopolymer paste) and two values of w/b ratio (0.35 and 0.38) were designed. Two series of geopolymers, specifically the L series (low w/b ratio) and H series (high w/b ratio), were measured in this research. All of the mixture proportions are presented in Table 3.

### 3.3. Mixing and Testing Procedure

**Figure 1:**
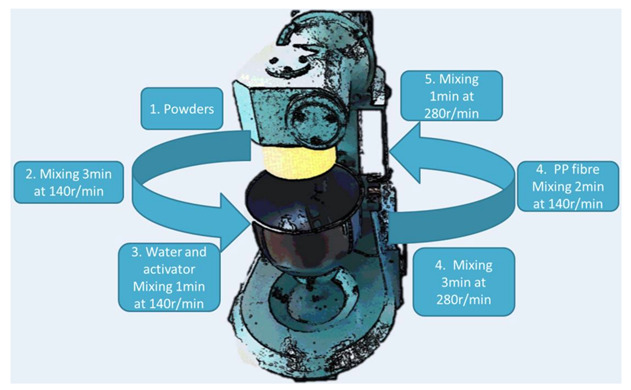
Mixing procedure of fibre-reinforced geopolymer paste.

The mixing procedure is shown in Figure 1. The workability of the fibre-reinforced geopolymer was assessed by determining the mini-slump flow and mini V-funnel flow time according to the European standard EFNARC [31,37]. The truncated cone measuring 60 mm × 70 mm× 100 mm was used herein. Slump spread test was carried out in two procedures. First, the truncated cone was placed on the centre of a flat steel plate and filled up by the paste. Second, the truncated cone was carefully lifted up vertically within five seconds, leaving the paste to spread freely and held for at least 3 min. Two perpendicular diameters of the paste patty formed after spread was measured and averaged as the slump spread value. The mini V-funnel flow time was defined as the time from the beginning of flowing to the first light coming into the funnel through the underneath outlet. Finally, the flow rate of the mixture was calculated by the ratio of mixture volume (1134 mL) and flow time with a unit of mL/s [31].

The specimens were demoulded 24 h after casting and were kept in a standard curing room with 20 ± 2 °C and humidity above 95% until being tested at 28 days. Flexural test (three-point bending) and compressive test were performed according to ISO 679-2009 [38]. Compressive strength was determined by the two portions of prisms left after the flexural test. Moreover, ultrasonic test and apparent density were directly associated with the porosity of specimens, which was mainly introduced by fibres.

## 4. Results and Discussion

### 4.1. Workabilities

**Figure 2:**
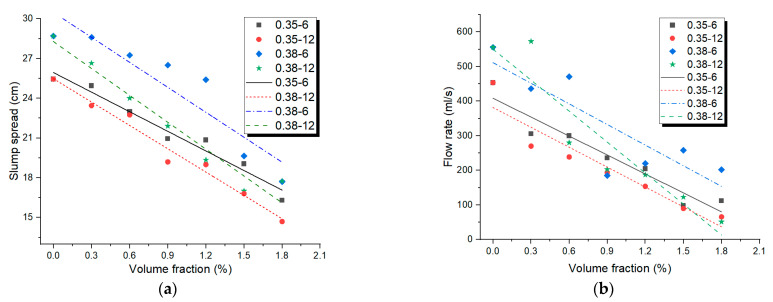
Relationships of (**a**) fibre volume fraction-slump spread; (**b**) fibre volume fraction-flow rate of PP fibre-reinforced geopolymer.

The relationship between slump spread and volume fraction of both 6 and 12 mm PP fibre is presented in Figure 2a. The regression study disclosed that there was a linear relationship between the volume fraction of fibre and the flowability of the geopolymer, shown as Equations (6)–(9). Additionally, the correlation coefficients between the two were very high, both above 0.8. It was similar with the results reported in literatures about PP fibre-reinforced Portland cementitious composites [28]. As anticipated, the slump spread was reduced by an increasing volume fraction of fibres. Hence, the slope values in Equations (6)–(9) are all negative values.

(6) $$w/b=0.38,\;\mathrm{fibre}\;\mathrm{length}=6\;\mathrm{mm}:SS=30.48-6.28V_{f},\;R^{2}=0.83$$

(7) $$w/b=0.38,\;\mathrm{fibre}\;\mathrm{length}=12\;\mathrm{mm}:\;SS=28.28-6.77V_{f},\;R^{2}=0.95$$

(8) $$w/b=0.35,\;\mathrm{fibre}\;\mathrm{length}=6\;\mathrm{mm}:\;SS=25.94-4.93V_{f},\;R^{2}=0.96$$

(9) $$w/b=0.35,\;\mathrm{fibre}\;\mathrm{length}=12\;\mathrm{mm}:\;SS=25.48-5.87V_{f},\;R^{2}=0.97$$

It was supposed that the declined flowability resulting from the using of fibres was prompted by the dealings of fibres, which resulted in internal resistance in contradiction of the flow. It is interesting that for a same volume fraction of fibre, the amount of shorter fibres was more than that of long fibres. However, the interactions between longer fibres were more active and so were the possibilities of fibre clumping or fibre balling, which could cause internal resistance against the flow. Hence, the absolute values of slope of Equations (7) and (9) were higher than those of Equations (6) and (8), respectively. This meant that the long PP fibres were more effective than short PP fibres in decreasing the flowability of the geopolymer. Similar results have been reported by Mehdipour et al. [29], who observed that, when only one type of glass fibre was used, mortars containing only shorter fibres had higher slump spread values than those with long fibres. However, it was opposite with the results about PP fibre-reinforced cementitious composites reported by Emdadi et al. [27]. This indicated that the mechanism of the PP fibre action in geopolymer and Portland cement-based composites may be different. In addition, the absolute values of slope of Equations (8) and (9) were higher than those of Equations (6) and (7), respectively. This indicated that in a high w/b matrix, the fibres were more effective in decreasing the flowability of the geopolymer. The main reason might be that in a matrix with a high water-binder ratio, the fibres could be better dispersed and, thus, play their roles more efficiently.

In the same manner, the relationship between the V-funnel flow rate and volume fraction of both 6 and 12 mm PP fibre is presented in Figure 2b, which was similar with the relationship between the slump spread and volume fraction. As can be seen, by increasing the fibre content, the viscosity of the geopolymer improved significantly and the addition of the long fibres had a more noteworthy influence on the flow. The regression study disclosed that there was a linear relationship between the volume fraction of the fibre and flow rate of the geopolymer, shown as Equations (10)–(13). Additionally, the correlation coefficients between the two were also good; most of them were about 0.9. It was similar with the results of slump spread, shown as Equations (6)–(9). Analogously, the flow rate was also reduced by increasing the volume fraction of PP fibres. Hence, the slope values in Equations (10)–(13) are all negative values, while the absolute values of slope of Equations (6)–(9) were much lower than those of Equations (10)–(13). This indicated that the PP fibre was more significant in decreasing the flow rate than decreasing the slump spread of the geopolymer. The difference between the slump flow spread and V-funnel flow rate results could be attributed to the free deformability and the restricted deformability of the flow properties that these tests measured, respectively.

(10) $$w/b=0.38,\;\mathrm{fibre}\;\mathrm{length}=6\;\mathrm{mm}:\;FR=511.34-198.6V_{f},\;R^{2}=0.73$$

(11) $$w/b=0.38,\;\mathrm{fibre}\;\mathrm{length}=12\;\mathrm{mm}:\;FR=550.34-298.45V_{f},\;R^{2}=0.89$$

(12) $$w/b=0.35,\;\mathrm{fibre}\;\mathrm{length}=6\;\mathrm{mm}:\;FR=408.69-182.47V_{f},\;R^{2}=0.92$$

(13) $$w/b=0.35,\;\mathrm{fibre}\;\mathrm{length}=12\;\mathrm{mm}:\;FR=381.84-191.62V_{f},\;R^{2}=0.90$$

**Figure 3:**
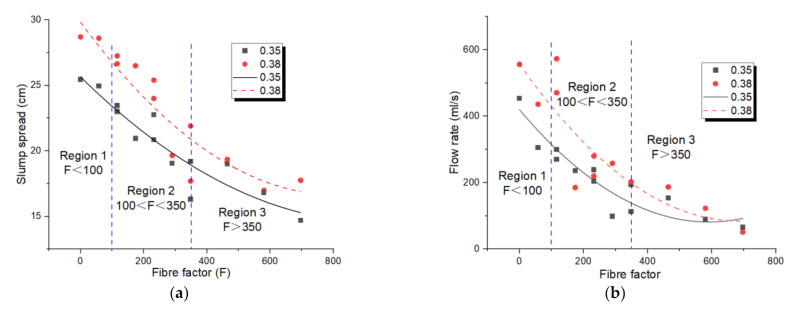
Relationships of (**a**) fibre factor–slump spread; (**b**) fibre factor–flow rate of PP fibre-reinforced geopolymer.

The consequences illustrated in Figure 2 present that both the length-to-diameter ratio and volume fraction of the PP fibres influenced the flowability properties of the geopolymer. Therefore, neither of these two factor was alone sufficient for evaluating the impact of fibres on fresh properties of the geopolymer. Based on the theory framework shown in the “2 Fibre Factor Theoretical methods” section, another parameter that was a combination of these two parameters was needed to accurately predict the effect of fibres on the workability of fresh mixtures. Thus, the product of these two factors, which is defined as the FF, has become a crucial factor in comparing different cementitious composites. Hence, Figure 3 illustrates the relationship between mini-slump flow spread and FF for the geopolymers with w/b = 0.35 and 0.38. In this regard, the results of mini-slump the flow spread and mini V-funnel flow rate against FF are shown in Figure 3. According to the illustrated theory in Section 2, the fibre-reinforced suspension incorporating fibre particles in the suspending fluid was classified into three diverse sections [31,32,33]:

In region one, where F < Fc = 100, the paste was considered as a dilute suspension and the fibre factor had a negligible or no impact on the flowability of the fibre-reinforced composites. In this region, the fibre particles were sufficiently far apart from each other, so the dealings of the fibres could be ignored. That is, as soon as the composite began to flow, the fibres began to acquire a favoured dispersal without any symbols of fibre clumping. For the geopolymer with a w/b of 0.38, the minimum slump spread of the geopolymers located in region one was 28.6 cm, which equals to that of the control group. Therefore, the flowability of suspension in this region was nearly the same as the flowability of suspending fluid (i.e., geopolymer paste).

In region two, where Fc = 100 < F < Fd = 350, composites were stiffer than its matrix paste and the addition of fibres began to impact on the flowability. As can be seen in Figure 3, growing the FF reduced both the mini slump flow spread and mini V-funnel flow rate of those mixtures located in region two. For the geopolymer with a w/b of 0.38, growing the FF from 0 to 116.13 and 348.39 in fibre-reinforced geopolymers reduced the flow rate by about −3% and 64%, respectively. According to the results, the worst flowability in region two belonged to the mix LSG6 with F = 348.39 (w/b = 0.35, volume fraction 1.8% and aspect ratio 193.55) in which the mini slump flow spread and mini V-funnel flow rate were 16.3 cm and 112.5 mL/s, respectively. As the FF was increased to near Fd = 350, the performance of the geopolymer was generally controlled by the contact network between the fibres. An observation during and after the tests showed that in both region one and region two, fibres were homogenously dispersed, without a significant instability or fibre balling and the geopolymers still had an adequate workability to flow freely by their own weight.

The flowability in region three F > Fd = 350 was absolutely changed. As the FF increased above 350, the flow features of the geopolymer reduced markedly and the composite was not able to flow spontaneously. As can be seen in Figure 3, fibres in region three were inclined to become tangled together and form knots; the poor fibre dispersion declined the flowability and stability of the geopolymer considerably. Based on the illustrated theory in Section 2 the fibre volume fraction of composites in this region was near the random close packing limit and the suspension became focused in which there existed a large amount of contact between fibres. In these cases, the growing of the flowability of the geopolymer may rise the risk of instability and worsen the overall workability of the composites.

The regression study disclosed that there was a quadratic function relationship between FF and the flowability of the geopolymer, shown as Equations (14)–(17). Additionally, the correlation coefficients between the two were very high, both about 0.9. It was similar with the results reported in literatures about the hybrid fibre-reinforced Portland cementitious composite [39]. As observed, the slump spread and flow rate were reduced by increasing FF. Then, the fresh composites could almost not flow, indicating that the quadratic function curve reached its lowest point.

(14) $$w/b=0.35:\;SS=29.82-0.03F+2.02F^{2},\;R^{2}=0.88$$

(15) $$w/b=0.35:\;FR=491.67-9.66F^{2},\;R^{2}=0.90$$

(16) $$w/b=0.38:\;SS=25.65-0.02F+1.26F^{2},\;R^{2}=0.90$$

(17) $$w/b=0.38:\;FR=557.29+1.38F+0.001F^{2},\;R^{2}=0.90$$

**Figure 4:**
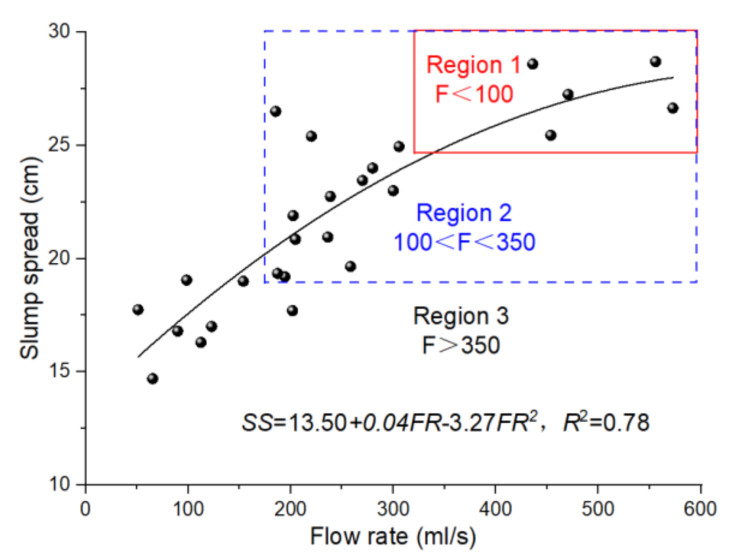
Relationship between slump spread and flow rate.

The relationship between the slump spread and V-funnel flow rate results are shown in Figure 4. The regression study disclosed that there was an up-leg of quadratic function relationship between the slump spread and V-funnel flow rate of PP fibre-reinforced geopolymer, shown as Figure 4. By growing the FF, the influence of fibre addition on the flow rate was more significant than the slump spread. This could be ascribed to the impact of fibre addition and rubbing on the flow rate assessed by the V-funnel. Hence, it can be concluded that the influence of fibres on the viscosity was more significant than the yield stress of the fibre-reinforced geopolymer. Mehdipour et al. [29] have reported that the slump spread test might not produce steady consequences in assessing the workability of glass fibre-reinforced cement paste. This can be ascribed to the experiment way, which only assessed the free deformability of the composite and could not professionally assess the constrained deformability, which was significant in the case of the fibre-reinforced composite. In the case of the fibre-reinforced composite, in addition to the flowability concern, the viscosity of the fresh composite may play a significant role in fibre dispersal, which impacts the fibre-toughing competence.

### 4.2. Mechanical Properties

**Figure 5:**
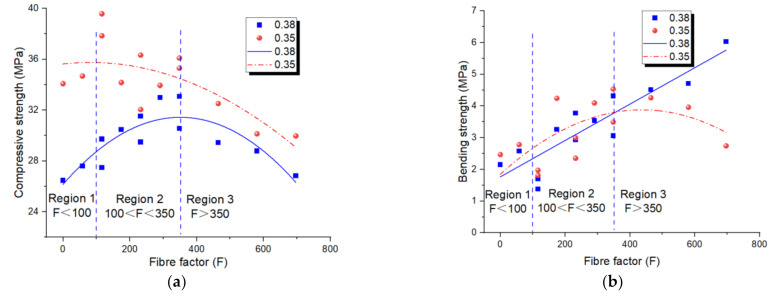
Relationships of (**a**) fibre factor–compressive strength; (**b**) fibre factor–bending strength of PP fibre-reinforced geopolymer.

Figure 5a shows the influence of FF on compressive strength. The results display that, for the w/b = 0.35 series, the compressive strength firstly rose (above 6%), but as soon as the FF touched F = Fd = 350, the compressive strength decreased. Namely, for a w/b ratio of 0.35 and FF less than 350, fibres were generally well dispersed and showed a uniform paste. Nevertheless, by growing the FF more than Fd, fibres tended to form clumpings and air could be ensnared in the composites and then prompt extra flaws in the geopolymer by inadequate compactness. Different consequences were illustrated in the literatures on the impact of fibres on the compressive strength of composites. The conflicting results in the literature could be expounded by the detail that, while the influence of fibres on the compressive strength was insignificant in the composite with an FF lower than the critical value Fc = 100, they had a significant influence on the composites with FF above the dense value Fd.

For an assumed fibre amount, nonetheless, compressive strength was predictable to fall with a growing w/b. In this case, in addition to the fibre balling, symbols of unsteadiness such as segregation and bleeding resulted in a higher decrease in the compressive strength. However, the compressive strength of the w/b = 0.38 series presented a significant increase with an FF below 350. In this case, an increase in FF does not damage the compressive strength of fibre-reinforced geopolymer. This can be ascribed to the good dispersion of fibres in the greater fluidable matrix. Hence, a low fibre content (F < 350) would not introduce many defects in the polymer. However, when FF increased above 350, the compressive strength of the geopolymer also decreased significantly, due to the extra flaws introduced by fibres.

The impacts of FF on the bending strength of the geopolymers are displayed in Figure 5b. As predicted, the adding of fibre presented a noteworthy influence on the bending strengths of geopolymers. PP fibre bridges crack under load and transfer the load, restricting of the development and conjoining of cracks. As the FF enlarged from 0 to 350, the bending strengths increased. However, for an FF above 350, the tendency conversed and the adding of fibres worsened the bending strength of geopolymers. The consequences of the workability disclosed that these geopolymers (F > 350) suffered from a poor flowability and were prone to fibre balling and non-uniform distribution. Hence, it should be noted that it cannot always be true that the higher the fibre amount in a composite the higher the growth in bending strengths. Consequently, fibre uniformity was one of the crucial elements to obtain the applicable mechanical properties. Moreover, according to the results, the long fibres were more effective under a bending load due to the higher bridging length. For the w/b = 0.38 series, the bending strengths of fibre-reinforced geopolymers were always increased with the increasing FF. This also ascribed to the good dispersion of fibres in the greater fluidable matrix. It seemed that the compressive strength was more sensitive to the flaws from fibre inclusion. While the fibres were more effective in bridging the cracks under the bending load than that under the compressive load.

### 4.3. Ultrasonic Wave Velocity and Density

**Figure 6:**
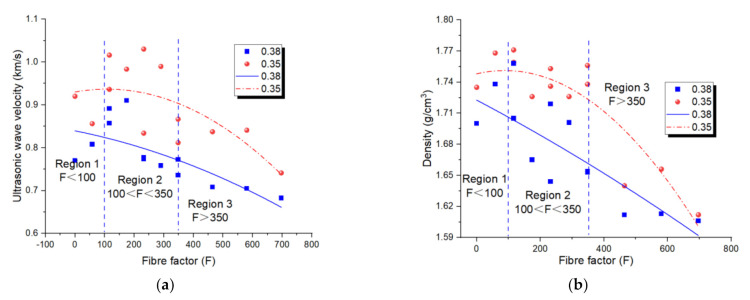
Relationships of (**a**) fibre factor-ultrasonic wave velocity; (**b**) fibre factor-density of PP fibre-reinforced geopolymer.

Figure 6a shows the relationship between the ultrasonic wave velocity and FF. Generally, the ultrasonic wave velocity of the geopolymer with a w/b ratio of 0.35 was higher than that of 0.38. The fall of ultrasonic wave velocity could be attributed to the existence of abundant free water in geopolymers. The ultrasonic wave velocity rose initially and then fell with the growth of FF, and the peak value of the ultrasonic wave velocity was achieved when 100 < FF < 350. The ultrasonic wave velocity revealed the compactness and homogeneousness of the composite. In regions one and two, the workability of the fibre-reinforced geopolymer tended to bridge the flaw and initial crack and, therefore, led to a dense geopolymer, so the highest value of ultrasonic wave velocity of geopolymer with two w/b ratios was achieved [33,40,41]. In region 3, the ultrasonic wave velocity fell with the growth of FF, which was an indication of a growth of flaws.

Figure 6b shows the relationship between the density and FF. It was clear that the variation tendency of the density of the PP fibre-reinforced geopolymer was similar with that of the ultrasonic wave velocity. Generally, the density of the geopolymer with a w/b ratio of 0.35 was higher than that of 0.38. The fall of density could be attributed to the existence of abundant free water in geopolymers. The density rose initially and then fell with the growth of FF, and the peak value of density was achieved when 100 < FF < 350. The density indicated a compactness and homogeneousness of the composite. In regions one and two, the workability of the fibre-reinforced geopolymer tended to bridge and fill the flaw and initial crack and therefore lead to dense geopolymer, so highest value of density of geopolymer with two w/b ratios were achieved [33,40,41]. In region 3, the density fell with the growth of FF, which was an indication of the growth of flaws. This also proved that the ultrasonic wave velocity could reflect the compactness of fibre-reinforced composites [42,43,44,45].

### 4.4. Further Discussion

**Figure 7:**
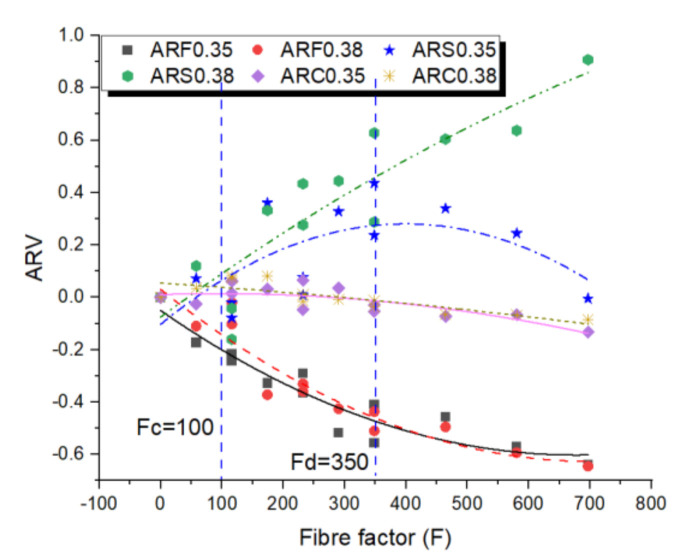
Influence of FF on average relative value of flowability, strength and compactness.

According to the acquired results, the fibre dispersal largely relies on the flowability of the composite as well as the volume fraction and aspect ratio of fibres in the composite. This conclusion is very useful because it offers an understanding for regulating the flowability of manufacturing self-compacting fibre-reinforced geopolymers to achieve a good fibre dispersal and, hence, applicable hardened properties. Consequently, to research the relationship between flowability and hardened properties, an average relative flowability (ARF), average relative strength (ARS) and average relative compactness (ARC) were designed. The ARF denotes the average relative slump spread and V-funnel flow rate of fresh geopolymers. ARS denotes the average of the relative compressive and bending strength of hardened geopolymers, while ARC denotes the average of the relative density and ultrasonic wave velocity of hardened geopolymers. The ARF, ARS and ARC against FF are illustrated as Figure 7. The consequences displayed that, for a volume fraction lower than Fc/(l/d) = 100/(l/d), the impact of fibres on properties of fibre-reinforced self-compacting geopolymer was negligible. Geopolymers in this region relate to stable and flowable composites without any symbols of fibre clumping or blocking. The inclusion of fibres considerably enhanced the bending strength of the fibre-reinforced geopolymers. The compressive strength of the fibre-reinforced self-compacting geopolymer was nearly the same as the compressive strength of the control group. This phenomenon could be described by the theory model illustrated in Section 2. Consequently, it was anticipated that by regulating the FF near Fc, the fibre-reinforced self-compacting geopolymer with an optimum flowability that improved the uniform fibre dispersal by the geopolymer and enhanced mechanical properties could be effortlessly reached.

While for the PP fibre volume fraction between Fc/(l/d) = Fc/100 and Fd/(l/d) = Fd/350, the performance of the geopolymer was powerfully controlled by the interaction linkage of the fibres and the composite changes from a dilute suspension into a semi-dilute suspension. Furthermore, a noteworthy enhancement of strengths (From 10% in region one to 65% in region two) was obtained in the price of a pronounced decline in flowability (from 20% in region one to 58% in region two). However, no symbols of instability and fibre clumping occurred of the geopolymer sited in this region. Consequently, if the best mechanical properties were needed, the FF could be carefully chosen to be higher than Fc; nonetheless, the engineer should be mindful about a predictable decrease in the flowability of a fresh composite. However, for geopolymer with a volume fraction higher than Fd/(l/d) = Fd/350, the non-uniform dispersal and balling of fibres occurred, resulting in a poor strength and compactness of geopolymers. In this region, for the w/b = 0.35 series, the average strengths were decreased from +40% in region two to 0% in region three and the geopolymers suffered from both a poor flowability and mechanical properties.

In view of both the flowability and mechanical properties, the Fc < FF < Fd was the ideal value of FF around which the geopolymers had good mechanical properties, but was still adequately workable to the flow by weight of self. Under this condition, a better fibre dispersal and reinforcing productivity could lead to better mechanical properties and compactness.

In the past 10 years, the influences of FF on the properties of Portland cementitious composites have been explored by some researchers [27,28,29,30]. Mehdipour et al. [29] presented that, for glass fibre, the Fc and Fd were 30 and 180, respectively. While for PP fibre, the Fc and Fd were 100 and 300, respectively [28]. Martinie et al. [30] presented that the Fd was 400 for steel fibre. Si et al. [33] illustrated that the Fc and Fd of PVA fibre were 100 and 400, respectively. Based on this research, the Fc and Fd of the PP fibre-reinforced geopolymer were 100 and 350, respectively. It was clear that this research reached close results compared with that of previous studies about PP or PVA fibre-reinforced Portland cementitious composites. Depending on the criterion provided by Martinie et al. [30], the rigidity index is defined as Equation (18).

(18) $$\frac{f}{l}≅\frac{\tau_{0}}{E}{\left(\frac{l}{d}\right)}^{3}$$

where *τ*_0_ is the yield stress of the composite, *E* is the modulus of elasticity and *l*/*d* is the aspect ratio of the fibres. Fibres with a rigidity index value lower than 1% are called rigid fibres [30]. The rigidity index value of the PP fibre in the geopolymer investigated herein ranged from 2.6% to 20.9%, which was higher than 1%. The rigidity indexes of steel fibre, glass fibre, PP fibre were 0.03%, 2%, 39–3500%, respectively [33]. The rigidity index of the PP fibre in the geopolymer investigated herein was close to the PP fibre in cementitious composites. Therefore, the PP fibre in the geopolymer investigated herein was a typical non-rigid fibre. The yield stress rose quickly when the FF surpassed 100, regardless of the fibre type. Consequently, it is practical accepting that the geopolymer composite behaviour changed considerably when the fibre volume fraction was higher than 100/(*l*/*d*) in respect to most non-rigid fibres.

## 5. Conclusions

Fibre self-compacting polymers have great potential in civil engineering applications, such as chord member grouting for concrete-filled steel tubular truss beams. However, to the best of the authors’ knowledge, the quantitative relationship between FF and the properties of the fibre-reinforced geopolymer has been rarely reported. In this research, 26 groups of mixtures were used to study the influence of PP FF on the flowability and mechanical properties and, also, the compactness of the fibre-reinforced self-compacting geopolymer. The conclusions are as follows:

(1) At the same volume fraction, geopolymers with long fibres presented a worse flowability than those having short fibres due to the easier contacting of long fibres.
(2) By the growing of FF, the influence of the fibre addition on the V-funnel flow rate was more significant than the slump spread. This could be ascribed to the consequence of fibre addition and friction by the V-funnel which estimated the restrained deformability.
(3) For an FF lesser than Fc = 100, the influence of fibres was negligible and fibres were far apart from each other and, thus, they could not restrict cracking under a load and transfer the load to improve the mechanical properties.
(4) For an FF between Fc = 100 and Fd = 350, a noteworthy enhancement of mechanical properties was obtained and the geopolymer was still adequately workable to flow by weight of self, without any symbols of instability and fibre clumping. Under this condition, a better fibre dispersal and reinforcing productivity can lead to better hardened properties.
(5) For FF higher than Fd = 350, fibres tended to come to be entwined together and formed clumping resulting from the fibre balling, resulting in worse hardened properties.

## Figures and Tables

**Table 1:** Constituents of mineral admixtures as wt.%.

Composition	CaO	SiO_2_	Al_2_O_3_	Fe_2_O_3_	MgO	K_2_O	SO_3_	Na_2_O
Fly ash	9.80	51.49	24.36	5.49	1.20	1.04	2.14	0.51
Slag	33.10	27.91	15.57	0.36	0.30	0.44	1.10	0.49
Silica fume	0.81	93.47	0.16	0.10	0.95	2.89	0.84	0.23

**Table 2:** Properties of the polypropylene fibre.

Length(mm)	Density (g/cm^3^)	Tensile Strength (MPa)	Diameter (μm)	Elastic Modulus (GPa)	Aspect Ratio (L/d)
6	0.91	530	31	5.0	193.55
12	0.91	530	31	5.0	387.09

**Table 3:** Mixture proportions of fibre-reinforced geopolymer.

Group	w/b	Aspect Ratio (L/d)	Volume Fraction (ϕ, %)	Fibre Factor (FF = L/d × ϕ)
LG	0.35	-	0.0	-
LSG1	0.35	193.55	0.3	58.06
LSG2	0.35	193.55	0.6	116.13
LSG3	0.35	193.55	0.9	174.19
LSG4	0.35	193.55	1.2	232.26
LSG5	0.35	193.55	1.5	290.32
LSG6	0.35	193.55	1.8	348.39
LLG1	0.35	387.09	0.3	116.13
LLG2	0.35	387.09	0.6	232.26
LLG3	0.35	387.09	0.9	348.39
LLG4	0.35	387.09	1.2	464.52
LLG5	0.35	387.09	1.5	580.65
LLG6	0.35	387.09	1.8	696.77
HG	0.38	-	0.0	-
HSG1	0.38	193.55	0.3	58.06
HSG2	0.38	193.55	0.6	116.13
HSG3	0.38	193.55	0.9	174.19
HSG4	0.38	193.55	1.2	232.26
HSG5	0.38	193.55	1.5	290.32
HSG6	0.38	193.55	1.8	348.39
HLG1	0.38	387.09	0.3	116.13
HLG2	0.38	387.09	0.6	232.26
HLG3	0.38	387.09	0.9	348.39
HLG4	0.38	387.09	1.2	464.52
HLG5	0.38	387.09	1.5	580.65
HLG6	0.38	387.09	1.8	696.77

## Data Availability

Data are available on request from the authors.
